# Exploring student perceptions on virtual reality in anatomy education: insights on enjoyment, effectiveness, and preferences

**DOI:** 10.1186/s12909-024-06370-6

**Published:** 2024-12-02

**Authors:** Mohammed Al-Hor, Hamad Almahdi, Majed Al-Theyab, Ayman G. Mustafa, Mohammed Seed Ahmed, Sami Zaqout

**Affiliations:** Department of Basic Medical Sciences, College of Medicine, QU Health, Qatar University, Doha, Qatar

**Keywords:** Virtual reality, Anatomy education, Medical education, Student perceptions, Blended learning

## Abstract

**Background:**

The dynamic landscape of medical education demands innovative teaching methods. This study introduces virtual reality (VR) technology to anatomy courses at Qatar University, aiming to assess students’ receptiveness to virtual anatomy dissection and its potential transformative impact.

**Methods:**

The study utilized a comprehensive survey and the 3D-Organon VR anatomy software to explore students’ perceptions and acceptance of VR in comparison with traditional anatomy learning tools during practical sessions. Fisher’s exact test for independence was performed to gauge shifts in students’ attitudes and preferences towards different educational modalities.

**Results:**

The findings reveal a generally positive reception towards VR, with many students indicating a preference for VR over traditional methods. The study noted significant improvements in understanding and memorization attributed to the use of VR. Exposure to a variety of educational modalities led to notable shifts in student perceptions, particularly an increase in positive perceptions regarding the understanding of anatomy lectures and a heightened preference for VR as a learning method.

**Conclusions:**

The study underscores the evolving and adaptable attitudes of students towards VR, emphasizing the significant role that diverse learning experiences play in shaping their receptiveness. It provides valuable insights into how medical education can be reshaped through a blended approach that integrates technological innovation with traditional learning methods. These findings advocate for the strategic incorporation of VR in anatomy courses to enhance learning outcomes.

## Background

Anatomy education plays a fundamental role in the global medical school curriculum, serving as the cornerstone for building a robust preclinical knowledge base vital for future physicians. A deep understanding of human anatomy is crucial for performing successful physical examinations, interpreting clinical symptoms, conducting surgeries, and undertaking a wide range of medical interventions [1–3]. Historically, the teaching of anatomical knowledge has predominantly revolved around cadaveric dissection. This traditional approach excels in its ability to unravel the intricacies of large organs, present three-dimensional bodily structures, showcase the spectrum of normal anatomical variations, and provide insights into clinically relevant aspects [4–6]. Advocates of traditional dissection passionately affirm its indispensability, emphasizing the irreplaceable value of hands-on learning [7, 8]. However, it is imperative to acknowledge that this conventional method is not without limitations. It falls short in effectively imparting certain complex anatomical concepts, such as surface anatomy, intricate details of small organs, the complexities of nerves, vessels, lymphatics, and various intricate aspects [4]. Moreover, for some students, the dissecting room becomes a source of stress and anxiety [9–11]. Recognizing these challenges, an ever-growing body of educators, students, and researchers has posited that sole reliance on dissection may not fully equip medical students to meet the multifaceted demands of modern healthcare [4, 12–14].

In response to these evolving considerations, a substantial shift has swept through the realm of medical education in recent years. This transformation is characterized by the embrace of computer-based and multimedia-assisted educational tools, encompassing videos, animations, three-dimensional models, and virtual microscopy, all designed to elevate the teaching of anatomy [8, 14–21].

In such a landscape, Extended Reality (XR) technologies have emerged as powerful tools for enhancing the learning experience, particularly in the field of anatomy education. XR is an umbrella term that encompasses various immersive technologies, including Virtual Reality (VR), Augmented Reality (AR), and Mixed Reality (MR), each offering unique ways to engage with and understand complex anatomical structures [22]. Among these, VR has gained significant attention as an innovative method for augmenting anatomy education.

To assess the efficacy of VR in anatomy education, a series of studies, as summarized in the provided abstracts, have been undertaken. These studies meticulously examine the utility of VR in comparison to traditional methods, including lectures and cadaveric dissection, consistently reflecting strong support for VR technology in enriching anatomical knowledge [23]. Furthermore, the convergence of digital anatomy and VR within medical training is poised to propel advances in healthcare practices, harnessing strengths and embracing opportunities, while vigilantly acknowledging limitations [24].

Several specific studies have spotlighted the enhanced understanding of anatomy achievable through VR-based methods, as evidenced by studies on heart anatomy [25] and improved test scores [26]. Additionally, the development of VR software tailored for cranial anatomy education underlines the technology’s potential applications in specialized domains [27]. It is of paramount importance to underscore the necessity for standardized implementation and comprehensive assessment of VR in medical education [28].

While the benefits of VR in anatomy education are well-established in the literature, the specific gap this work addresses is the application of VR tools, such as 3D-Organon, in unique educational and cultural settings. Notably, this study is the first to explore the implementation of VR in anatomy teaching at Qatar University. Our primary aim is to measure the receptiveness of these students to virtual anatomy dissection, thereby illuminating the transformative potential of this innovative approach. This study is particularly focused on establishing norms for learning preferences and evaluating the perceived effectiveness of 3D-Organon VR anatomy software within the context of anatomy courses. To achieve this, we delve into students’ perceptions and acceptance of VR technology, making comparisons with traditional learning tools such as plastic models and the Anatomage table, an advanced virtual dissection system, during anatomy practical sessions. In addition, our investigation delved into discerning any significant distinctions between students who utilized VR before and after engaging with other educational modalities. This comparison was integral to understanding the varied impacts of VR in the context of different educational sequences. Ultimately, our research strives to offer valuable insights into how VR can reshape medical education, enhancing the educational experiences of future physicians.

## Methods

### Study population

Participants for this study were drawn from Year-1 to Year-4 students in the College of Medicine (CMED) at Qatar University enrolled in anatomy lab courses during the 2023/2024 academic year. All students were asked to voluntarily participate in the study after reading and signing a consent form approved by the IRB committee at Qatar University (No. 1844-EA/23). In regular anatomy lab sessions, students used a variety of learning modalities, including plastic/plastinated models and the Anatomage table. As part of the study, an additional optional station featuring VR Oculus headset devices was introduced. Students were given a structured opportunity to explore the anatomical structures relevant to the designated lab session, focusing on specific body systems.

### Study design

The primary objective of this study was to compare the perceived effectiveness of VR education models to standard anatomy education methods in enhancing students’ understanding of human anatomy. Additionally, the study aimed to assess students’ attitudes toward virtual anatomy dissection using 3D-Organon VR anatomy software, in comparison to their attitudes toward regular anatomy labs. To evaluate these aspects, an anonymous questionnaire was utilized.

Each lab session lasted two hours and focused on a specific anatomical system. During these sessions, students had the opportunity to explore the corresponding anatomical structures using VR, alongside traditional learning tools such as the Anatomage table and plastic models. This alignment allowed for direct comparisons between the different modalities.

Students were given 5 min to familiarize themselves with the VR headsets and software before proceeding. Following this brief orientation, they were given 10 min to explore the relevant anatomical structures using the VR devices.

### Data collection and analysis

The questionnaire was distributed in a paper-based format to the students after they had used the various learning modalities in the lab. The collected data were processed and analyzed to generate descriptive statistics and identify correlations. The study focused on comparing the experiences of students who used VR before engaging with other educational modalities to those who used VR after other modalities. Fisher’s exact test for independence was used to rigorously examine associations between variables, given its appropriateness for categorical data analysis. All statistical analyses were conducted using GraphPad Prism V.9.

## Results

**Fig. 1:**
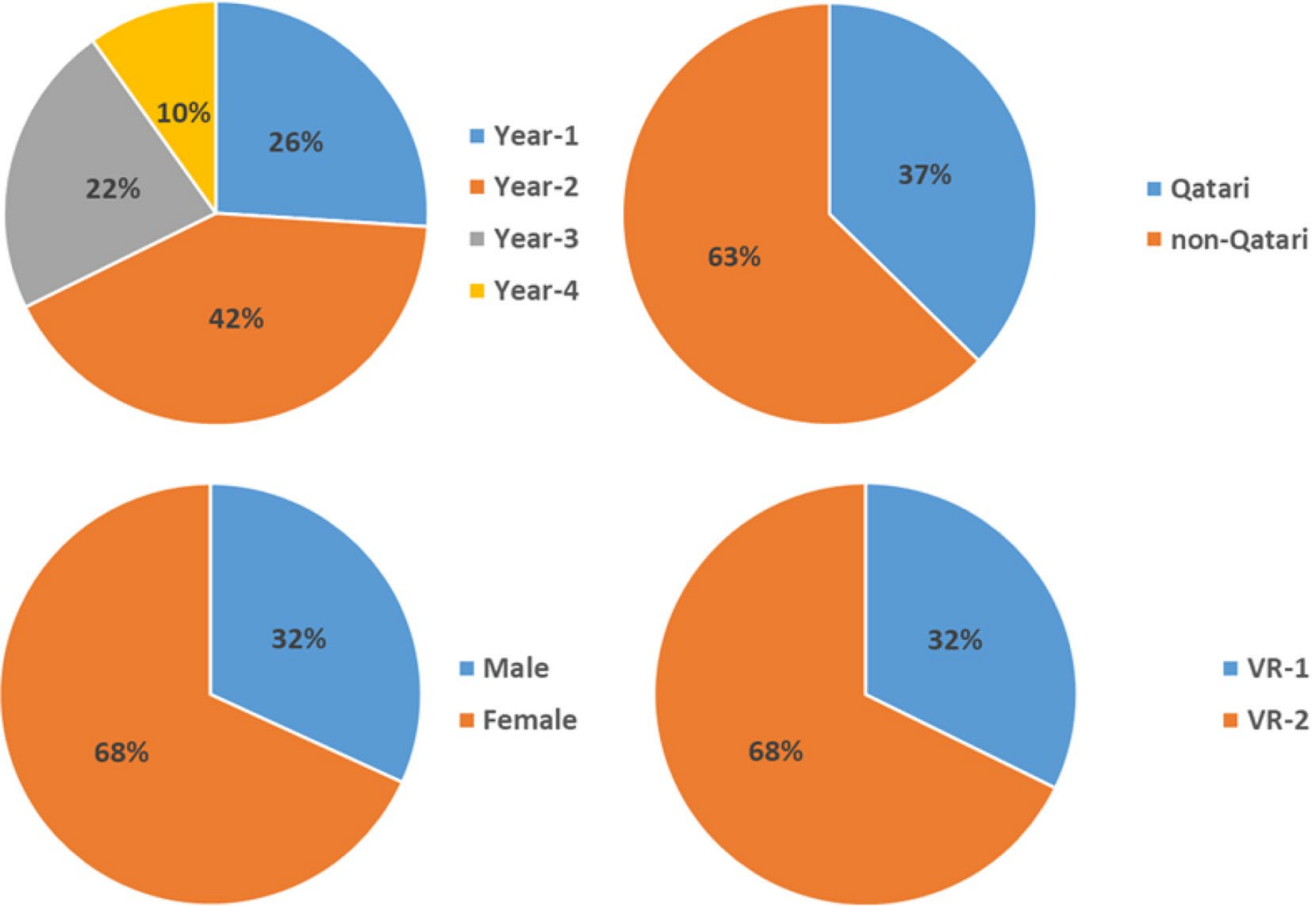
Background information and characteristics of the study sample (*n* = 223). **VR-1** represents the percentage of students who utilized VR before engaging with other educational modalities (*n* = 72). VR-2 represents the percentage of students who utilized VR after engaging with other educational modalities (*n* = 151)

The study enrolled 223 participants across various academic years, spanning Year-1 to Year-4, as depicted in (Fig. 1). The mean age of participants stood at 19.3 years. In terms of gender distribution, the participants included 71 male students (31.8%) and 152 female students (68.2%). Furthermore, the demographic composition reflected 83 national Qatari students (37.2%) and 140 non-Qatari students (62.8%). Notably, the majority of non-Qatari students were from Egypt (15%), Jordan (12.1%), Syria (11.4%), and Pakistan (9.3%).

### Overall student perceptions on VR

#### Enjoyment and learning

**Fig. 2:**
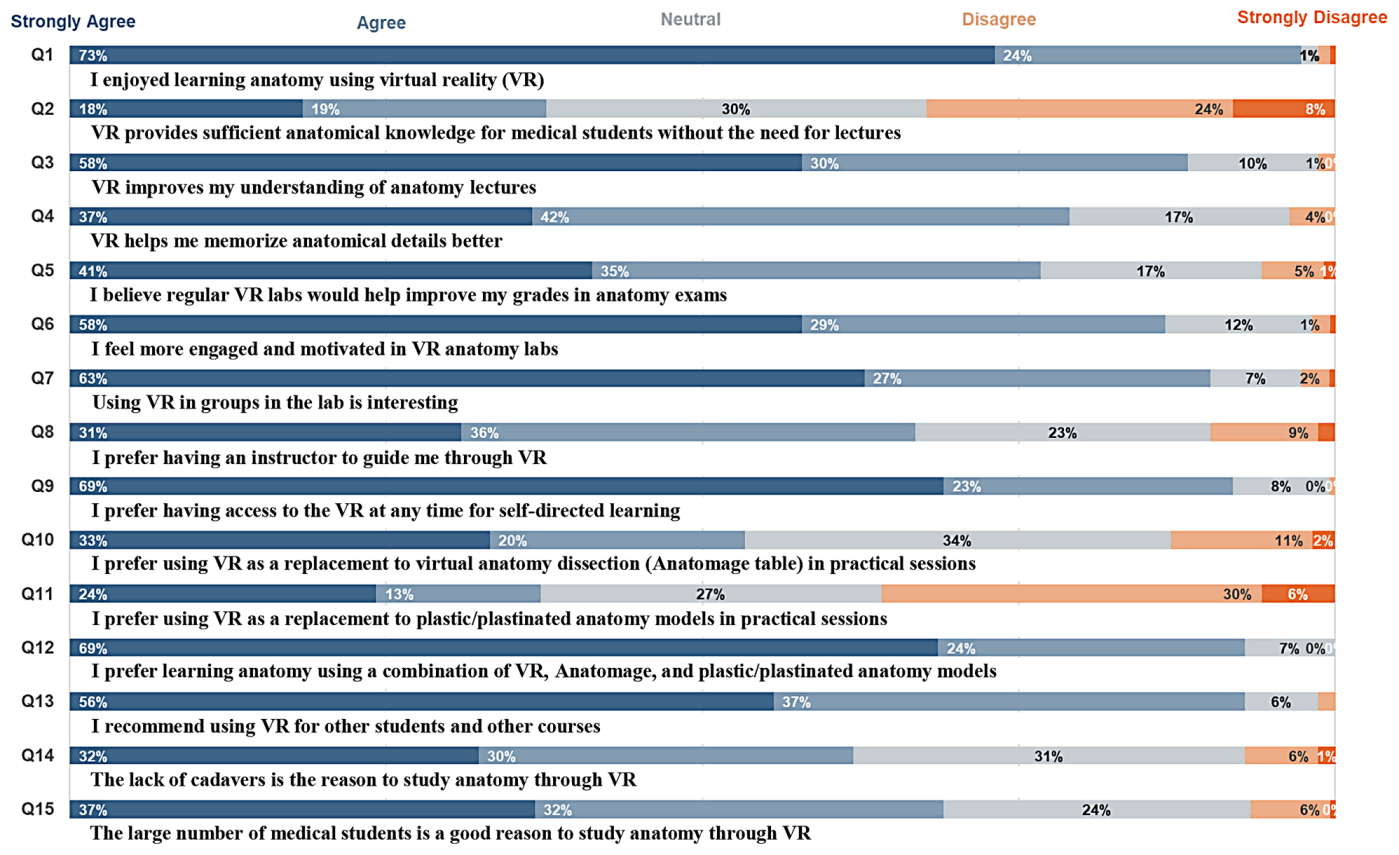
Overall student perspectives on VR

73% of respondents strongly agreed that they enjoyed learning anatomy through VR, underscoring a notably positive reception of VR technology in this educational domain (Q1, Fig. 2).

#### Effectiveness of VR

Opinions varied regarding the sufficiency of VR for anatomical knowledge in the absence of traditional lectures. Responses indicated a spectrum of viewpoints, with 18% strongly agreeing, 19% agreeing, and 30% remaining neutral. In contrast, 24% disagreed, and 8% strongly disagreed (Q2, Fig. 2).

#### Enhancement of understanding

When assessing VR’s impact on comprehending anatomy lectures, a majority − 58% strongly agreed and 30% agreed - acknowledged that VR significantly improves their understanding (Q3, Fig. 2).

#### Memorization and academic performance

While 37% of respondents strongly agreed that VR aided in better memorization of anatomical details, 42% agreed, and 17% remained neutral (Q4, Fig. 2). Furthermore, 41% strongly agreed that regular VR labs could potentially bolster their grades in anatomy exams, with 35% in agreement (Q5, Fig. 2).

#### Engagement and motivation

The exploration of engagement and motivation levels in VR anatomy labs revealed that 58% strongly agreed, 29% agreed, and 12% remained neutral (Q6, Fig. 2).

#### Preferences in learning environment

Findings suggested a strong inclination towards group-based VR learning, with 63% strongly agreeing, 27% agreeing, and 7% remaining neutral (Q7, Fig. 2). Additionally, 31% strongly agreed, 36% agreed, and 23% were neutral in their preference for an instructor-guided VR experience (Q8, Fig. 2). A significant 69% strongly agreed that having unrestricted access to VR for self-directed learning was preferable, with 23% in agreement (Q9, Fig. 2).

#### Preference for replacing traditional methods

Opinions diverged concerning the substitution of traditional anatomy education methods with VR technology. Notably, 33% strongly agreed, 20% agreed, 34% were neutral, 11% disagreed, and 2% strongly disagreed regarding replacing virtual anatomy dissection (Anatomage table) with VR (Q10, Fig. 2). Similarly, 24% strongly agreed, 13% agreed, 27% were neutral, 30% disagreed, and 6% strongly disagreed regarding replacing plastic/plastinated anatomy models with VR (Q11, Fig. 2). A substantial 69% strongly agreed, 24% agreed, and 7% were neutral on preferring a combined approach utilizing VR, Anatomage, and plastic/plastinated anatomy models (Q12, Fig. 2).

#### Recommendation and reasons

We found that 56% strongly agreed, 37% agreed, 6% were neutral, and 1% disagreed that they would recommend the use of VR for other students and courses, particularly in medical fields such as radiology and pathology, where visualizing both radiological and pathological changes can significantly enhance learning (Q13, Fig. 2). Regarding reasons for studying anatomy through VR, 32% agreed, 30% were neutral, 31% disagreed, 6% strongly disagreed, and 1% remained uncertain (Q14, Fig. 2).

#### Consideration of class size

Regarding the impact of class size on anatomy education, 37% strongly agreed, 32% agreed, 24% were neutral, 6% disagreed, and none strongly disagreed when considering the large number of medical students as a motivation to study anatomy through VR (Q15, Fig. 2).

### Navigating Student preferences

#### Preferences in anatomy learning methods

**Fig. 3:**
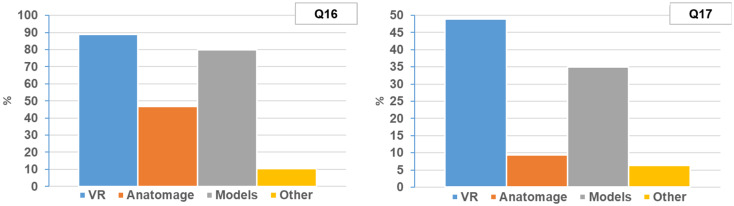
Overall students’ preferences in anatomy learning methods. **Q16**- I prefer learning anatomy using the following methods (multiple selections were allowed). **Q17**- My favorite method for learning anatomy is

Respondents expressed their preference for learning anatomy through various methods. The majority favored VR (88.8%), followed by plastic/plastinated models (79.8%), Anatomage (46.6%), and other methods (10.3%), primarily citing cadavers and mobile apps (Q16, Fig. 3).

#### Favorite method for learning anatomy

When asked to choose their favorite method for learning anatomy, VR garnered the highest preference at 48.9%, followed by plastic/plastinated models (35.0%), Anatomage (9.4%), and other methods (6.3%), notably including cadavers and textbooks (Q17, Fig. 3).

### VR before vs. after other educational modalities

In examining the impact of engaging with other educational modalities on students’ perceptions of VR in anatomy education, several noteworthy trends emerged.

#### VR’s impact on understanding anatomy lectures

A significant difference was observed when comparing the responses between the group that was exposed to VR prior to engaging with other educational modalities (such as Anatomage and plastic/plastinated anatomy models, referred to as VR1) and the group that was exposed to VR after using these modalities (VR2). Prior to engaging with other modalities, 46% of students in VR1 strongly agreed that VR improved their understanding of anatomy lectures, whereas 64% in VR2 expressed a strong agreement. This shift suggests an increased positive perception in understanding anatomy lectures after exposure to additional educational modalities (Q3, Table 1).

**Table 1:**
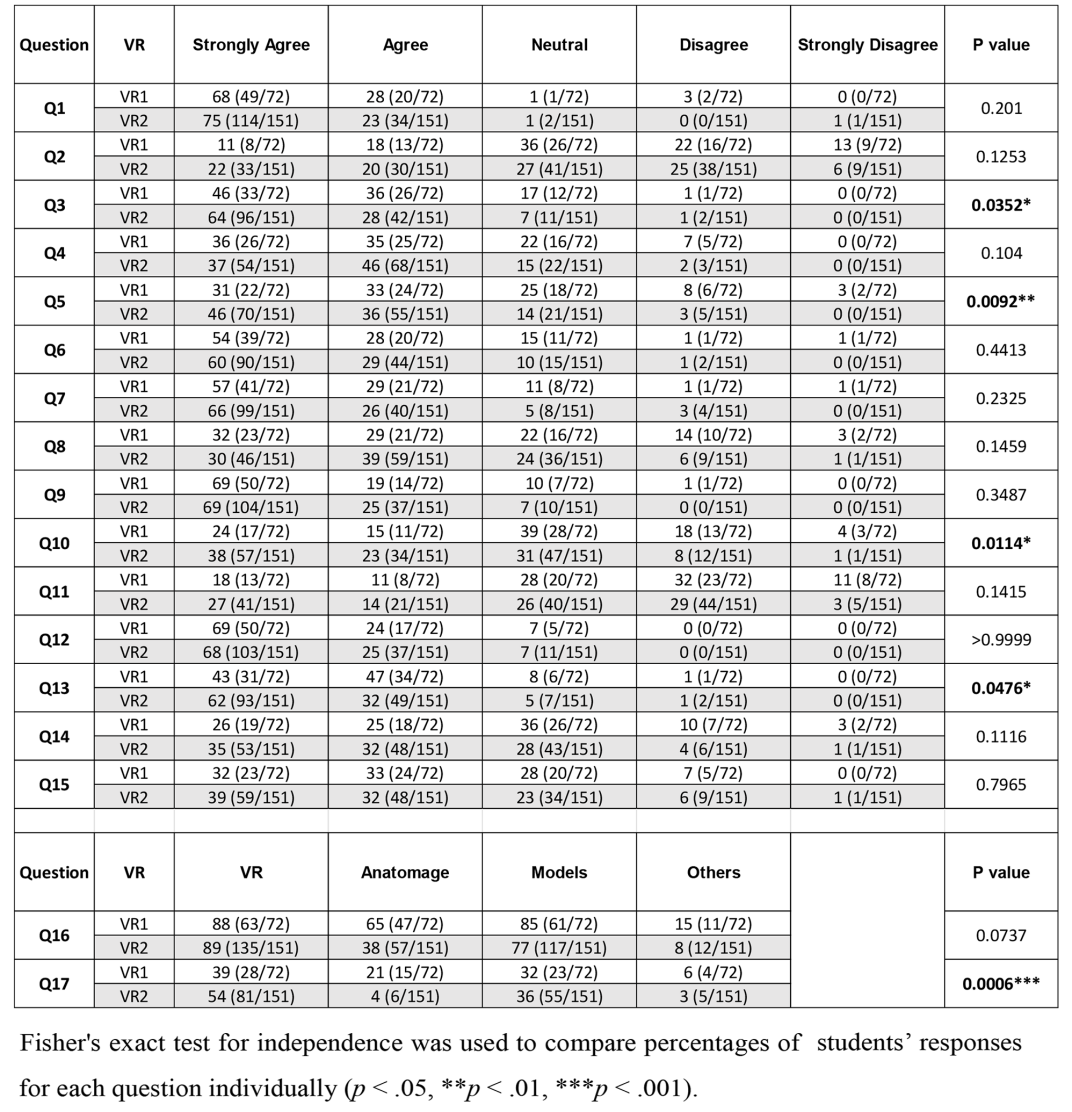
Students’ responses between VR before (VR1) and after (VR2) exposure to additional educational modalities

#### Expectations on VR labs and academic performance

In assessing beliefs regarding the impact of VR labs on academic performance, a noticeable change was observed. In VR1, 31% strongly agreed that implementing more VR lab sessions into the students’ weekly class schedules would help improve grades, compared to 46% in VR2. This suggests an increased positive expectation regarding the contribution of VR labs to academic performance after exposure to other educational modalities (Q5, Table 1).

#### Preference for VR in practical sessions

Students’ preferences for VR as a replacement in practical sessions exhibited a shift. In VR1, 24% strongly agreed, while 38% in VR2 expressed a strong agreement. Conversely, the percentage of students neutral or in disagreement decreased after exposure to additional educational modalities, indicating a shift in preference towards using VR as a replacement (Q10, Table 1).

#### Recommendation of VR for other students and courses

When considering students’ willingness to recommend VR, the data indicated a notable change. In VR1, 43% strongly agreed to recommend VR, while in VR2, 62% expressed a strong agreement. This implies an increased likelihood of recommending VR to other students and courses after exposure to additional educational modalities (Q13, Table 1).

#### Preferred method for learning anatomy

Examining preferences for learning anatomy, the shift in favor of VR was evident after engaging with other educational modalities. In VR1, 39% preferred VR, whereas in VR2, this percentage increased to 54%. This shift underscores a heightened preference for VR as a learning method after exposure to additional educational modalities (Q17, Table 1).

## Discussion

The primary focus of this study was to assess how medical students perceive and engage with VR technology within the realm of anatomy education. Through a comprehensive survey comprising 17 questions, we aimed to probe into the diverse attitudes and opinions of students, covering various aspects of VR implementation in anatomy education. The resulting insights illuminate the landscape of student preferences and attitudes toward the integration of VR into their anatomy learning experiences.

### Integration of VR in anatomy education

The integration of VR into medical education, particularly in anatomy instruction, represents a paradigm shift from traditional teaching methods. This study aligns with the broader trend in medical education, reflecting a departure from exclusive reliance on cadaveric dissection towards a more diversified approach that incorporates technology-enhanced learning. The limitations of traditional dissection methods, including challenges in conveying certain anatomical concepts and the emotional stress experienced by students, have been well-documented in the literature. Dissection, while beneficial for surgical skill development and understanding whole-body pathology [4], has also been associated with significant emotional and psychological stress, particularly among students unfamiliar with cadavers or unprepared for the dissecting room experience [9–11]. The stress experienced in these environments, ranging from intrusive thoughts to symptoms resembling post-traumatic stress disorder (PTSD), underscores the need for supplementary educational modalities [11]. These findings highlight the potential value of integrating VR as an adjunct to traditional methods.

### Comparison with previous research

Several studies mentioned in the introduction consistently support the idea that VR technology contributes positively to anatomical education [23]. The observed positive reception of VR in this study, evidenced by the majority expressing enjoyment (73%), improved understanding (58%), and better memorization (79%), aligns with findings from studies focusing on specific anatomical areas like heart anatomy [25]. The endorsement of VR by the majority of students reflects a trend observed in other studies emphasizing the effectiveness of VR in enhancing test scores [26].

### Effectiveness and limitations of VR

While the positive feedback on enjoyment and learning is encouraging, opinions on the sufficiency of VR as a standalone tool for anatomical knowledge were more diverse. This aligns with the existing discourse in the literature that acknowledges the benefits of VR but also calls for a balanced approach that integrates it with traditional methods [4, 12–14]. The study underscores the importance of considering a combined approach, recognizing that VR, while beneficial, may not entirely replace traditional methods.

### Preferences and learning environment

The preference for group-based VR learning (63%), instructor-guided experiences (67%), and unrestricted access for self-directed learning (69%) highlights the nuanced nature of student preferences. This aligns with literature emphasizing the significance of a student-centered, flexible learning environment in medical education [29, 30]. The study’s findings also resonate with the broader discourse on the importance of collaborative and guided learning experiences in the context of VR [31, 32].

### Substitution of traditional methods

The study delves into students’ willingness to replace traditional methods with VR, revealing varied opinions. The inclination to replace traditional methods, such as virtual anatomy dissection (67%) and plastic models (57%), with VR suggests a readiness for technological integration. However, a significant portion of students remains neutral, emphasizing the need for a balanced approach that caters to diverse preferences. This aligns with literature advocating for a thoughtful and gradual integration of technology into medical education [33, 34].

### Impact of exposure to educational modalities

The notable shifts in student perceptions after exposure to additional educational modalities highlight the dynamic nature of attitudes towards VR. The increased positive perception in understanding anatomy lectures (Q3) and the heightened preference for VR as a learning method (Q17) after exposure to other modalities underscore the potential influence of varied learning experiences on students’ views.

### Recommendation and future directions

The majority’s willingness to recommend VR for other students and courses (93%) suggests a positive outlook on the technology’s broader applicability. The study contributes to the literature by emphasizing the importance of understanding students’ perspectives in shaping the future of medical education. Future research could explore the long-term impact of VR integration, consider faculty perspectives, and investigate the optimal balance between traditional and technological approaches in anatomy education [24, 28].

## Conclusions

In conclusion, this study adds valuable insights to the discussion on integrating VR into anatomy education. The positive reception of VR by medical students and the diversity of opinions emphasize the need for a flexible approach. Exposure to alternative educational methods proves influential in shaping students’ favorable views of VR, extending beyond mere reception to impact overall perceptions, preferences, and expectations in anatomy education. The findings stress the importance of a blended approach that combines technological innovation with traditional teaching methods, highlighting the significance of adaptability in shaping the future of anatomy education.

## Data Availability

No datasets were generated or analysed during the current study.
